# Sad as a Matter of Evidence: The Desire for Self-Verification Motivates the Pursuit of Sadness in Clinical Depression

**DOI:** 10.3389/fpsyg.2020.00238

**Published:** 2020-02-19

**Authors:** Elisabeth A. Arens, Ulrich Stangier

**Affiliations:** Department of Clinical Psychology and Psychotherapy, Goethe University Frankfurt, Frankfurt, Germany

**Keywords:** major depressive disorder, emotion regulation, desired affect, goals, motives

## Abstract

**Background:**

Research on desired emotions revealed that individuals want to feel negative emotions if they expect these emotions to yield certain benefits. In previous studies, the pursuit of sadness (e.g., via pursuing art that evokes sadness) has been attributed to hedonic motives, i.e., to feel pleasure. We propose that in individuals with major depressive disorder (MDD) the pursuit of sadness may be more strongly related to self-verification motives, i.e., to sustain their sense of self through feeling sad.

**Methods:**

Participants with MDD (*n* = 50) were compared to non-depressed controls (*n* = 50) in their desired emotional states, as indicated by selected music (sad, happy and neutral), and in their motives (hedonic vs. self-verification) for choosing sad music. Groups were also compared in their self-reported general preference for sadness and the perceived functionality of sadness.

**Results:**

MDD participants showed a significant higher desire for sadness; more than half of them deliberately chose sad music. Whereas MDD participants had a marked preference for self-verification over hedonic motives, the reverse was true for non-depressed controls. MDD participants also agreed more strongly with self-verifying functions of sadness and expressed a stronger general preference for sadness.

**Conclusion:**

Findings indicate that emotion regulation in MDD might be driven by self-verification motives. They point to the relevance of exploring patients’ desired emotional states and associated motives. The systematic integration of positive affect into the self-image of depressed patients might help to deemphasize the self-verifying function of sadness, thereby overcoming the depression.

## Introduction

Major depressive disorder (MDD) is characterized by intense and frequent negative emotions, especially persistent sadness (American Psychiatric Association [APA], 2013). It seems obvious that individuals suffering from MDD make every effort to reduce such aversive emotional states in order “to feel better.” Paradoxically, however, individuals with depressive symptoms were shown to actively maintain their feelings of sadness, e.g., by exposing themselves voluntarily to sad stimuli (e.g., music, pictures) (Garrido and Schubert, 2013; Millgram et al., 2019, 2015). Why would individuals with MDD deliberately choose affective states that contribute to their own unhappiness?

From a hedonic perspective on emotion regulation (e.g., Larsen, 2000) desired affective states are determined by short-term hedonic motives, i.e., to maximize pleasure and to minimize pain (Higgins, 2014). Following the approach of Huta and Ryan (2010), hedonia is the pursuit of feeling good and relaxation. Indeed, when asked what they want to feel and why, participants listed hedonic motives (e.g., “I want to feel good”) as underlying their attempts on 50% of the cases (Augustine et al., 2010). Paradoxically, also the deliberate choice of sad media (e.g., music, movies etc.) has been attributed to hedonic motives, i.e., to the experience of pleasure (e.g., Oliver, 1993; Schramm and Wirth, 2010; Huron, 2011), also referred to as “hedonic reversal” (Rozin et al., 2013; for a review see Sachs et al., 2015). A recent study of Yoon et al. (2019) supports the assumption that also depressed individuals’ choice of sad music is motivated by hedonic aims. When asked for reasons for their sad music preference, most participants with MDD freely reported that the sad music had a calming effect on them. However, some characteristics of the study sample raise questions related to the generalizability of the findings. The authors investigated a rather young (mean age 20 years), exclusively female student sample. Given that MDD has a mean age of onset ranging from 25 to 45 years (Kessler et al., 2007), and a lower but still considerable proportion of men (Kendler and Gardner, 2014), the representativeness of this sample seems limited.

A contrasting view on emotion regulation holds that seeking certain affective states serves not only hedonic but also instrumental motives (Bonanno and Burton, 2013; Tamir, 2016). Whereas *hedonic* motives target the immediate phenomenological value of emotions, *instrumental* motives in emotion regulation target potential values of emotions other than their immediate phenomenology (e.g., to feel good). According to this instrumental approach to emotion regulation, sometimes individuals will postpone fulfilment of hedonic motives and will be motivated to activate/maintain negative emotions as long as they expect a desirable benefit (Tamir, 2016). In fact, previous research has reported that individuals perceive the deliberate engagement with sad stimuli (esp. music) as functional, for instance because they perceive it as helpful coping strategy. In a study of Van den Tol et al. (2016), individuals reported that listening to sad music helps them to get in touch with and expressing affect. Furthermore, through the re-experiencing of affect, listening to sad music was shown to be associated with acceptance coping. Similarly, two large-scale surveys in which participants were asked to provide their motives for listening to sad music have revealed that sad music is associated with understanding feelings and emotional assurance (Taruffi and Koelsch, 2014) and engaging in a process of catharsis (Garrido and Schubert, 2011). However, individuals with clinical depression were not included in these studies and perceived benefits of music-evoked sadness might substantially differ in healthy vs. clinically depressed individuals.

It has been assumed that the deliberate engagement with sad stimuli of individuals with depression may contribute to a self-consistent self-perception (Millgram et al., 2015; Tamir, 2016). According to the theory of self-verification (Swann, 2012) individuals have a general preference for information consistent with the beliefs about themselves (Swann and Schroeder, 1995). Because chronic self-concepts play an important role in understanding the world, providing a sense of coherence, and guiding action, individuals become motivated to maintain those concepts through self-verification. Such strivings provide stability to people’s lives, making their experiences more coherent (Talaifar and Swann, 2017). Accordingly, individuals may be motivated to experience emotions that provide self- or belief-consistent information, whether such beliefs are positive or negative (see Jansz and Timmers, 2002). Indeed, a negative self-concept was shown to predict self-reported experience of negative affect (Conner and Barrett, 2005). As depression is associated with a negative self-construction (Sowislo and Orth, 2013), individuals with MDD may have a self-concept that incorporates negative affect.

Research on self-verification theory has shown that having one self’s view verified is reinforcing, even when that self-view is negative (Swann et al., 1987; Giesler et al., 1996; Swann, 2012; Ayduk et al., 2013; Brummelman et al., 2016).

Some indirect evidence supports the assumption that depressed individuals engage with sad stimuli for self-verifying reasons. For instance, individuals with depression were shown to use sad music to engage in rumination (i.e., to intensify negative affect and to focus on negative thoughts and memories) (Garrido et al., 2017). Likewise, individuals with tendencies to depression expected that listening to sad music will benefit them in some way, for instance because it makes them think about past events in their life (Garrido and Schubert, 2015). In application of the self-verification theory to these findings, the selective processing of negative information seen in rumination might serve to verify depressed people’s views of themselves and this verification could be highly reinforcing (Nolen-Hoeksema et al., 2008). In a study of Wilhelm et al. (2013) participants were asked about their reasons for listening to music. The degree to which depressed participants referenced engaging with music in order to “express, experience, or understand emotions” was significantly higher than in healthy controls. According to the self-verification literature an important way to reveal one’s true self is to express emotions consistent with one’s current psychological and emotional state. In another study, people with low self-esteem were more likely than people with high self-esteem to dampen positive mood (Wood et al., 2003) and less likely to repair sad moods (Wood et al., 2009), partly because such moods were more familiar to them. Because depression is linked to low self-esteem (e.g., Orth and Robins, 2013) depressed people may similarly be motivated to experience sadness to verify their emotional selves.

In sum, studies on the pursuit of sadness with a special focus given to clinical depression have revealed somewhat contradictory findings. Whereas one study (Yoon et al., 2019) suggests that listening to sad music in depressed individuals is related to hedonic benefits (i.e., calming effects), some indirect evidence (e.g., Wilhelm et al., 2013; Garrido et al., 2017) suggests that the deliberate engagement with sad music is related to instrumental benefits (i.e., a verifying self-perception).

In light of these contradicting findings, we aimed to conduct a study that extends our understanding of motives underlying the deliberate choice of sad music in depressed individuals. To contrast hedonic vs. self-verification motives, we included an assessment containing two reasons for choosing sad music, one targeting the *hedonic* motive (“I have chosen the music clip because it made me feel good”) and one targeting the *self-verification* motive (“I have chosen the music clip because it made me feel like myself”). Note that this was not a forced-choice format, as both response options were rated on a six-point Likert-type scale. Thus, we expected this study to provide clear evidence whether listening to sad music is related to hedonic motives and/or self-verification motives or to none of both. In addition, we assessed participants’ perceived general functionality of sadness, i.e., whether sadness is generally perceived as self-verifying or hedonic emotion. We also assessed the general desire to experience sadness (vs. happiness). As depressed individuals were shown to use music to match or reflect mood (Wilhelm et al., 2013) we controlled for concurrent emotions, in order to rule out mood-congruency effects.

We expected to replicate the findings of Millgram et al. (2015) and Yoon et al. (2019), demonstrating that individuals with MDD choose more frequently sad music compared to non-depressed controls. Based on previous studies, we further expected that motives for choosing sad music would differ in depressed vs. non-depressed individuals. In light of the above-mentioned contradicting findings, we did not specify the direction of the expected difference. Finally, we tested whether perceiving sadness as self-verifying or hedonic emotion in general would be positively correlated with the choice of sad music and the general preference for sadness.

## Materials and Methods

### Participants

A total of fifty individuals with current MDD (mean age 41.37, *SD* = 13.79; 56% female) were recruited from the outpatient clinic of the Goethe University Frankfurt. Clinical status was confirmed in a separate laboratory session, administering the Structured Clinical Interview for DSM-IV Axis I Disorders (SCID-I; First et al., 1997). Patients were excluded for history of bipolar disorder, alcohol or substance abuse, and psychotic disorders. 50 non-depressed control individuals (mean age 41.10, *SD* = 15.81; 66% female) were recruited via advertisements in local newspapers and social media. A short telephone interview screened for current symptoms of affective disorders. Participants were included if they had no history of any affective disorder, alcohol or substance abuse, and psychotic disorders, assessed with the SCID-I Interview following the telephone screening. As expected, depressed individuals scored significantly higher on the Beck Depression Inventory (Beck et al., 1961) (*M* = 26.35, *SD* = 5.83) than non-depressed controls (*M* = 3.520, *SD* = 4.047), *t*(98) = −14.088, *p* = 0.000. Groups did not differ in age, *t*(98) = −0.082, *p* = NS; sex, χ*^2^*(1, *N* = 100) = 4.453, *p* = NS or educational level, χ*^2^*(3, *N* = 100) = 5.081, *p* = NS. All participants received 35$ for participating.

### Instruments and Procedure

After the assessment of participants’ clinical status, they were invited for a second session including the music-selection task. The two sessions were conducted approximately one week apart (3–10 days, *M* = 7.53 days). Participants gave informed consent and rated the extent to which they currently felt happiness and sadness (1 = very little, 6 = extremely). Then participants completed the music-selection task (Millgram et al., 2015), in which they listened to six short excerpts (30 sec.) of sad, happy and neutral music-clips in random order. The task included two sad music clips (“Adagio for Strings” by Samuel Barber; “Rakavot” by Avi Balili), two happy music clips (“Track 8” by Jay Hannah; “Infernal Galop” from *Orpheus in the Underworld* by Jacques Offenbach), and two neutral music clips (“Pickles” by Edgar Meyer; “First Thing” by Four Test). In order to rule out music lyrics as confounding variable, clips contained solely instrumental music. For each emotional valence (sad, happy, neutral), there were two clips covering both genres (classical and modern), leading to a total of six clips. Clips were pretested in a healthy German sample (*n* = 24) to ensure that they induce the emotional reaction they are supposed to. After listening to the short excerpts, participants were asked to select one clip they wanted to listen to in its entirety later in the experiment. Selections were made by entering the respective number the clip had in the sequence (1–6).

Music choice indicated the desired affective state. After having selected one clip, participants answered two short questions on a six-point Likert-type scale about possible motives of their choice, one targeting the *hedonic* motive: “I have chosen the music clip because it made me feel good” and one targeting the *self-verification* motive: “I have chosen the music clip because it made me feel like myself.” Questions were drawn from a questionnaire used by Augustine et al. (2010) measuring motives for desired affective states. Afterward, participants rated the extent to which each clip made them feel sad and happy (1 = not at all, 9 = extremely) (manipulation check).

To assess participants’ general preference for sadness (vs. happiness), we followed previous studies that assessed motivation to experience emotions (Kim et al., 2015; Tamir et al., 2016). Participants rated the degree to which they generally wanted to experience happiness and sadness in daily life (1 = *not at all*, 7 = *extremely*; e.g., “Please indicate the extent to which you generally want to feel happiness in your daily life”). To assess the degree of motivation to experience happiness, we averaged across ratings of happy, glad, joyful, light-hearted, and cheerful (α = 0.76). To assess the degree of motivation to experience sadness, we averaged across ratings of depressed, melancholic, downhearted, sad, and gloomy (α = 0.83). The construct and predictive validity of this measure for assessing the degree of motivation to experience emotions has been established (e.g., Hackenbracht and Tamir, 2010; Tamir et al., 2013; Millgram et al., 2015; Porat et al., 2016).

Questions for the assessment of the perceived functionality were newly developed but based on existing theoretical accounts. Following the approach of Huta and Ryan (2010), hedonia includes the pursuit of feeling good and relaxation which are assumed to promote a sense of subjective vitality (Ryan and Frederick, 1997). Accordingly, participants’ perceived hedonic function of sadness was measured using three items each of them relating of one of these aspects: *Sadness can make*… *1.me feel calm and relaxed; 2.feel good; 3.me feel alive* (α = 0.71).

Following the definition of self-verification of Talaifar and Swann (2017), individuals aim to make their true self visible to others and confirm their stable self-views. Accordingly, participants’ perceived self-verification function of sadness was measured using the following three items: *Sadness can make*… *1.me be more like the person I truly am; 2.me feel confirmed in my self-perception; 3.my true self visible* (α = 0.80).

## Results

As manipulation check we ran a repeated measures ANOVA with group (depressed, non-depressed) as between-subjects factor and music valence (happy, neutral, sad) and emotional rating (happiness, sadness) as within-subjects factors. The analysis yielded a significant main effect of music valence, *F*(2,98) = 43.42, *p* < 0.001, η^2^ = 0.31, indicating that the emotional ratings to the neutral music clips (*M* = 3.19) were weaker than the emotional reactions to the sad music clips (*M* = 5.28), *p* = 0.002 and the happy music clips (*M* = 5.84), *p* = 0.008.

As expected, we found a significant music type × emotional rating interaction, *F*(2,97) = 126.20, *p* < 0.001, η*^2^* = 0.72. Follow-up tests revealed that sad music clips received higher ratings of sadness (*M* = 5.18, *SD* = 2.15) than the happy (*M* = 1.44, *SD* = 1.01), *p* < 0.001, and the neutral music clips (*M* = 2.72, *SD* = 1.63), *p* = 0.003. Likewise, the happy music-clips received higher ratings of happiness (*M* = 5.87, *SD* = 2.07) than did the sad (*M* = 3.02, *SD* = 1.09), *p* < 0.001, and the neutral music clips (*M* = 3.33, *SD* = 1.88), *p* = 0.001. The group × emotional rating × music valence interaction was not significant, *F*(2,98) = 0.50, *p* = 0.673, η^2^ < 0.01, which indicates that depressed and non-depressed participants did not differ in their reactions to the sad music clips or to the happy music clips.

In order to examine whether MDD group status predict a greater likelihood of preferring sad music, a multinomial logistic regression was conducted, with group (0 = non-depressed, 1 = depressed) as the independent variable and selected music valence (1 = happy, 2 = neutral, 3 = sad) as the dependent variable. Groups differed significantly in their selection of music clips, χ*^2^*(2, *N* = 100) = 6.34, *p* = 0.042. Compared to controls, depressed participants choose more frequently sad music than happy music, *b* = 1.13, Wald χ*^2^* (1) = 5.86, OR = 3.09, *p* = 0.015, and more frequently sad music than neutral music, *b* = 2.14, Wald χ^2^(1) = 7.56, OR = 6.10, *p* = 0.021. Table 1 presents the percentages of depressed and non-depressed participants who selected each type of music-clip. There was no effect of state sadness, χ*^2^*(2, *N* = 100) = 4.72, *p* = 0.095, or state happiness χ*^2^*(2, *N* = 100) = 3.21, *p* = 1.019. Compared to the control group, depressed participants were still more likely than to select sad music than to select happy, *b* = 0.48, Wald χ*^2^*(1) = 3.27, OR = 1.89, *p* = 0.035, and neutral music, b = 0.37, Wald χ*^2^*(1) = 2.90, OR = 1.39, *p* = 0.049.

**TABLE 1:** Descriptive statistics, group comparisons and correlations among the key variables.

	Depressed (*n* = 50)	Non-depressed (*n* = 50)	Correlations^a^			
			
			1	2	3	4
1. Music-clip selected,%						
1 = happy	33	50	–			
2 = neutral	16	18				
3 = sad	51	32				
2. Self-reported preference for sadness (DAS), mean (SD)	1.4 (0.6)	1.1 (0.2)	0.27**	_		
3. Self-reported preference for happiness (DAS), mean (SD)	4.1 (0.6)	4.2 (0.5)	–0.06	−0.46***	_	
4. Perceived hedonic function of sadness, mean (SD)	3.3 (1.5)	3.5 (1.1)	0.32*	0.07	0.05	_
5. Perceived self-verifying function of sadness, mean (SD)	3.1 (1.2)	1.8 (0.8)	0.32*	0.27**	−0.18*	–0.02

^*a*^ Correlations are computed across the whole sample; point-biserial correlations were computed for the categorical variable (music-clip selected), variable was dichotomized with 1 = happy music, 2 = sad music; for the continuous variables (self-reported preferences for sadness, self- reported preferences for happiness, perceived hedonic function of sadness, and perceived self-verifying function of sadness) Pearson correlations are presented.* *p* ≤ 0.05; ** *p* ≤ 0.01; *** *p* ≤ 0.001.

**FIGURE 1:**
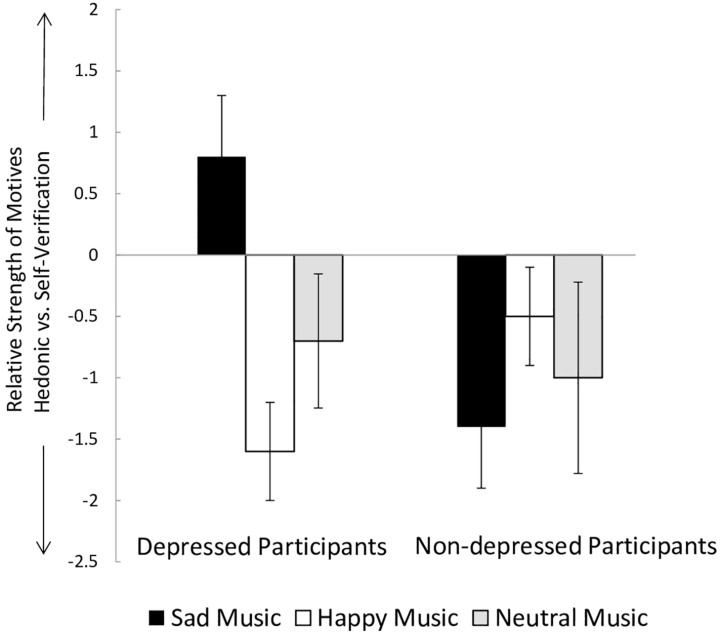
Difference scores^a^ between self-verification and hedonic motives for choosing sad, happy and neutral music in depressed (*n* = 50) and non-depressed (*n* = 50) participants. ^a^ Scores were calculated by subtracting mean values of hedonic motive from mean values of self-verification motive; positive values indicate a relative preference for self-verification motive, negative values indicate a relative preference for hedonic motive.

In order to directly contrast preferences for superordinate motives of music choice, we computed difference scores by subtracting the mean value of the hedonic motive measure from the mean value of the self-verification motive measure for each subject; thus higher positive scores represent a favor of the self-verification motive over the hedonic motive (and vice versa). Two-sample *t*-tests were computed with group (depressed ns non-depressed) as independent variable and difference score as dependent variable. Those depressed participants who chose a sad music clip, reported more frequently a self-verification motive to be the reason of their choice, compared to non-depressed participants, who primarily reported a hedonic motive, *t*(21) = −3.99, *p* < 0.001, *d* = 1.34 (see Figure 1). In contrast, those depressed participants who chose happy music, more clearly stated a hedonic motive as reason than non-depressed participants, *t*(40) = 2.69, *p* = 0.010, *d* = 0.86. Groups did not differ in their motive for choosing a neutral clip, *t*(15) = −0.33, *p* = 0.098 In order to correct for multiple comparisons, we applied a Bonferroni correction and adjusted the significance level. All significant *t* scores remained significant.

To test differences in the general preference for affective states, we conducted a repeated measures ANOVA with group (depressed, non-depressed) as between-subjects factor and desired affective state (sadness, happiness) as within-subjects factor. We found a main effect of preference for affective state *F*(1,98) = 905.72, *p* < 0.001, η*^2^* = 0.901, indicating that all participants reported a stronger preference for happiness than for sadness. There was also a significant group × desired affective state interaction, *F*(1,98) = 3.86, *p* = 0.050, η*^2^* = 0.07. Follow-up tests confirmed that the preference for a negative affective state was significantly higher among depressed participants than among control participants, *F*(1,98) = 5.74, *p* = 0.019, η*^2^* = 0.06 (see Table 1).

A repeated measures ANOVA with group (depressed, non-depressed) as a between-subjects factor and perceived functions of sadness (hedonic vs. self-verification functions) as a within-subjects factor, yielded a significant main effect of perceived function, *F*(1,98) = 24.339, *p* < 0.001, η*^2^* = 0.19; on average, there was a stronger agreement in favor of perceived hedonic function of sadness (*M* = 3.45) than of self-verification function (*M* = 2.46). There was also a significant group × perceived function interaction, *F*(1,98) = 13.88, *p* < 0.001, η*^2^* = 0.12. Follow-up tests indicated that groups differed in their perceived functions of sadness. Depressed participants showed a significant higher agreement that sadness can have a self-verifying function than non-depressed controls, *F*(1,98) = 37.96, *p* < 0.001, η*^2^* = 0.28. There was no significant group difference in the perceived hedonic function of sadness, *F*(1,98) = 0.34, *p* = 0.562, η*^2^* = 0.01 (see Table 1).

In order to test whether perceiving sadness as self-verifying or hedonic emotion in general is positively associated with the choice of sad music and the general preference for sadness we computed Pearson correlations (for music selection as categorical variable point-biserial correlations were computed). Self-reported preference for sadness was positively correlated with the selection of sadness-inducing music, providing evidence for the convergent validity of our measures and suggests that both measures may reflect a motivation to experience sadness. Perceived self-verifying function, but not perceived hedonic function was positively correlated with self-reported preference for sadness (see Table 1).

## Discussion

The current results confirm previous findings that individuals suffering from MDD choose to maintain or even increase their sadness rather than trying to alleviate it (Werner-Seidler et al., 2013; Millgram et al., 2015; Yoon et al., 2019). Why do depressed patients seem to have a kind of a “motivational maintenance system,” i.e., a system that fosters the intention to remain fixed in the accustomed negative patterns of thinking and feeling? A more recent conception of the expectancy-value approach (Fishbein and Ajzen, 1975) assumes that not only behaviors, but also emotional states are associated with expectations regarding their reward value. According to this assumption, individuals would also be motivated to activate/maintain negative emotions as long as they expect a desirable benefit (Tamir, 2016). In line with this proposal, the current findings show that depressive participants attributed a significantly stronger instrumental benefit to the feeling of sadness than did controls, in particular a stronger approval of self-verification functions of sadness. Thus, whereas non-depressed controls were shown to acquire some hedonic benefit from sadness, which is consistent with previous reports of a hedonic-driven deliberate consumption of sad stimuli in the general population (e.g., Rozin et al., 2013), depressed subjects seem to be driven by the need of preserving a stable self-concept, regardless of any hedonic gain. Those quality differences in motives might make the desire for sadness more harmful in depressed than in healthy individuals, as they may be more persistent. Whereas the hedonic driven consumption of sad media might occur on an occasional basis that does not impact the affective well-being in the long run, consumption driven by self-verification motives may occur systematically as sad stimuli might be constantly sought out to stabilize the negative self-schema. This is supported by the current finding that the perceived self-verifying functionality of sadness was positively correlated with the self-reported preference for sadness, whereas this was not the case for the perceived hedonic function. As a negative self-view predicts depressive symptomatology (Montesano et al., 2017), exposure to sad stimuli in depressed individuals may contribute to a malignant course of the disorder, as this may serve to deepen self-limiting and negative beliefs about oneself. However, the causal role of the pursuit of sadness in the course and recovery from depression was not tested in this study. Prospective, longitudinal studies are needed to establish a causal link.

The findings of the current study are in contrast to the results of the study of Yoon et al. (2019), supporting the hedonic hypothesis in the deliberate choice of sad stimuli. There are several potential reasons for the conflicting findings. First, sample characteristics of the Yoon study were quite different to the current investigation, as they examined a quite young, exclusively female sample. Since younger age has been shown to be associated with different reasons for listening to music (compared to older age) (Groarke and Hogan, 2016) this may have led to diverging findings. We argue that the current findings supporting the instrumental hypothesis may have a greater generalizability as our sample was more representative in terms of age and gender of individuals with depression (see, e.g., Kessler et al., 2007; Kendler and Gardner, 2014). Second, Yoon et al. used an open-ended-question, whereas our results reflect the agreement with two closed-ended questions. Although open-ended questions have a range of methodological benefits, they also have some disadvantages, e.g., responses can be biased by articulateness and reflection-skills (Hyman and Sierra, 2016). Although speculative, we argue that hedonic reasons might be easier reflected than self-verification reasons, as the latter additionally require a reflection of the self-image. Thus, conflicting findings might mirror different levels of self-reflection. Thus, it is possible that participants in the Yoon study perceived sad music as calming *because* it is self-verifying but were just not aware of this higher-order-motive. This assumption is supported by the current finding that depressed participants agreed to some extent with the hedonic item but showed a relative stronger preference for the self-verification-item.

What are the implications for the treatment of depression? Although speculative, the findings presented here suggest that cognitive-behavioral treatment of emotion regulation deficits in depression should not work on a solely *competence* level, e.g., by practising more adaptive emotion regulation techniques. Rather, it seems more relevant to first address cognitive mechanisms in depression on a *motivational* level, e.g., by systematically exploring patients’ desired affective states (“how do I want to feel?”) and to identify instrumental motives behind desired affective states (“are feelings of sadness helpful to me, what function do they have for me?”). Rief and Glombiewski (2016) have pointed to the role of depression-typical negative expectations about the self-concept as common barrier to change. Mindfulness- and loving kindness-based interventions may be effective encouraging and integrating positive affect into the self-image of depressed patients. This could contribute to deemphasize the self-verification function of sadness, thereby helping to overcome depression. However, due to the study design, it is not possible to establish a causal link between self-verification motives and the pursuit of sadness. Therefore, it is not clear whether changing the self-image will necessarily impact the pursuit of sadness and thereby the course of depression. Further longitudinal psychotherapy research might explore the relevance of the integration of positive affect into the self-image for treatment response and outcome in depression.

Several additional limitations of the current study need to be acknowledged and taken into account when looking at clinical and theoretical implications of the results.

First, we did not include a control group beyond non-depressed subjects, e.g., patients exhibiting disorders commonly comorbid to MDD (e.g., anxiety disorders). This would allow to more carefully tease apart group differences directly attributable to MDD diagnosis. Second, most of our measures relied on self-report, so that we cannot rule out the possibility that data may have been affected by social desirability bias and/or perceived situational demands. Future research should apply an experimental study design, in which participants are unaware that their motives for desired affective states are measured. Third, the music selection task applied in this study solely included instrumental music. As music listening choices of many individuals in real life may frequently include music with lyrics, this might limit ecological validity of the current findings. Future studies, including also music with lyrics are needed in order to raise ecological validity of the study setting. However, care should be taken to control for lyrics as potential confounding variable. Fourth, we measured only two potential motives using single questions as output measures. Other studies have suggested that sadness might have also have other functions, e.g., social (Hackenbracht and Tamir, 2010) or eudaimonic (Oliver and Raney, 2011) functions. Although we focused primarily on the direct comparison of self-verification and hedonism, note that there was no forced-choice format, as both response options were rated on a six-point Likert-type scale. Thus, participants had the option to disagree with both items. Given that there was a high mean agreement with the self-verification item (*M* = 5.01), these findings suggest that, regardless to its comparison to hedonic motives, self-verification might play a pivotal role in the deliberate choice of sad music in depressed individuals. Future studies, measuring several types of motives, are needed in order to further extend our understanding of the underlying motivation of the pursuit of sadness in clinical depression.

## Data Availability Statement

The datasets generated for this study are available on request to the corresponding author.

## Ethics Statement

The studies involving human participants were reviewed and approved by the Ethikkommission des Fachbereichs 05, Goethe Universität Frankfurt. The patients/participants provided their written informed consent to participate in this study.

## Author Contributions

Both authors developed the study concept and design, drafted the manuscript, approved the final version of the manuscript, and agreed to be accountable for all aspects of the work. EA performed the data collection and analysis.

## Conflict of Interest

The authors declare that the research was conducted in the absence of any commercial or financial relationships that could be construed as a potential conflict of interest.
